# Time series data of a broadleaved secondary forest in Japan as affected by deer and mass mortality of oak trees

**DOI:** 10.3897/BDJ.5.e11732

**Published:** 2017-03-17

**Authors:** Hiroki Itô

**Affiliations:** 1 Hokkaido Research Center, Forestry and Forest Products Research Institute, Sapporo, Japan

**Keywords:** broadleaved secondary forest, conifer plantation, deer impact, mass mortality of oak trees

## Abstract

**Background:**

Abandonment of broadleaved secondary forests that have been used for various purposes may cause the loss of biodiversity. Some of these forests suffer from diseases such as Japanese oak wilt. An increasing number of deer also impact some of them. Monitoring and recording the status of such forests is important for their proper management.

**New information:**

This data set provides a concrete example of temporal changes in a temperate broadleaved secondary forest. The forest has been damaged by mass mortality of oak trees caused by Japanese oak wilt disease. In addition, the forest has been under foraging pressure by sika deer (*Cervus nippon* Temminck). The data set can provide information on how such a forest has changed in species composition of the canopy and sub-canopy layers and in species occurrence in the understory layer.

## Introduction

The progress of succession due to underuse or abandonment of secondary forests that have been used for various purposes, such as sources of firewood and charcoal wood, may cause loss of biodiversity in Japan (Washitani 2001, Takeuchi 2010) as well as Europe (Rackham 2008, Müllerová et al. 2015). For example, the loss of mosaic land use maintained by human activity can cause habitat loss for various species which depend on the mosaic (Washitani 2001). Some of these forests suffer from diseases. In Japan, the mass mortality of oak trees caused by Japanese oak wilt disease severely affected oak-dominant secondary forests (Kuroda et al. 2012, Nakajima and Ishida 2014). The pathogen is a fungus, *Raffaelea quercivora* Kubono & Shin.Ito, which is carried by a specific beetle, *Platypus quercivorus* Murayama (Kubono and Ito 2002, Kinuura and Kobayashi 2006). Though the pathogen and the vector are both native to Japan, abandonment of the secondary forest in recent decades may be related to the epidemic (Nakajima and Ishida 2014).

In addition, deer impact also affects broadleaved forests in Japan (Takatsuki 2009) and other regions of the world (Côté et al. 2004, Gerhardt et al. 2013). In Japan, an increased number of sika deer (*Cervus nippon* Temminck) has impacted many plantations and natural forests (Takatsuki 2009). Their foraging can suppress regeneration of trees except for deer-unpalatable species (Itô 2015, Itô 2016).

It is important to monitor and record the changes in such forests to manage them properly. This data set provides information on how succession has proceeded in a broadleaf secondary forest in Japan and how the combined effects of the mass mortality of oak trees and deer impacts altered the forest. The data set consists of changes in species composition, stem density and stem diameter at breast height in the canopy and sub-canopy layer from 1993 to 2014, and changes in occurrence of woody species in the understory layer from 1992 to 2014.

## General description

### Purpose

This study was initiated in 1992 to monitor the dynamics of broadleaved secondary forests adjacent to urban areas. Although the study site also contained a conifer plantation, regeneration of broadleaved species within the plantation was also monitored. After periodic surveys (1993, 1996, 1999, 2002 and 2005) were completed (Itô 2007), mass mortality of oak trees affected the forest, alongside deer impacts. To evaluate the compound effect of both types of damage, the study site was surveyed again in 2014 (Itô 2015, Itô 2016).

## Sampling methods

### Study extent

This study was conducted in the Ginkakuzi-san (also spelled Ginkakuji-san) National Forest, Kyôto City, Japan. This area is located in the warm-temperate zone. The dominant vegetation of the area had been evergreen oak forest approximately 7000 to 1000 years ago, but secondary forests composed of pines and deciduous oaks increased after that (Takahara 2015). In the late 19th century, the forest around the study site was estimated to be covered with small pines, *Pinus densiflora* Siebold & Zucc., due to human impact (Ogura 2015). After nationalization (in the 1870s), the forest has been protected from felling as a rule. In the 1930s, a mixed forest of pines and deciduous tree species such as *Quercus serrata* Murray covered the site (Osaka Regional Forest Office 1936). However, the forest has not been completely free from felling; e.g., trees were cut for fuel wood during World War II, and part of the forest was converted to conifer (*Cryptomeria japonica* (L.f) D.Don and *Chamaecyparis obtusa* (Siebold & Zucc.) Endl.) plantations in the 1970s. Recently, broadleaved evergreen trees, such as *Symplocos prunifolia* Siebold & Zucc. and *Ilex pedunculosa* Miq., were thinned in some parts of the forest, probably to improve light penetration into the forest. Forest diseases also affected the forest. After the 1960s, pine wilt disease strongly affected the national forest and numerous *P. densiflora* trees died. The responsible pathogen is the nematode *Bursaphelenchus xylophilus* (Steiner & Buhrer) Nickle, which is thought to have been brought from North America (Mamiya 1988). In the last decade, mass mortality of oak trees affected the forest, as described above. In addition, increased numbers of sika deer have affected the forest (Itô 2015, Itô 2016). Though the population density of the deer in the forest is unknown, camera trap data showed that they inhabited the forest in all seasons (Itô 2015).

### Sampling description

**Figure 1.**
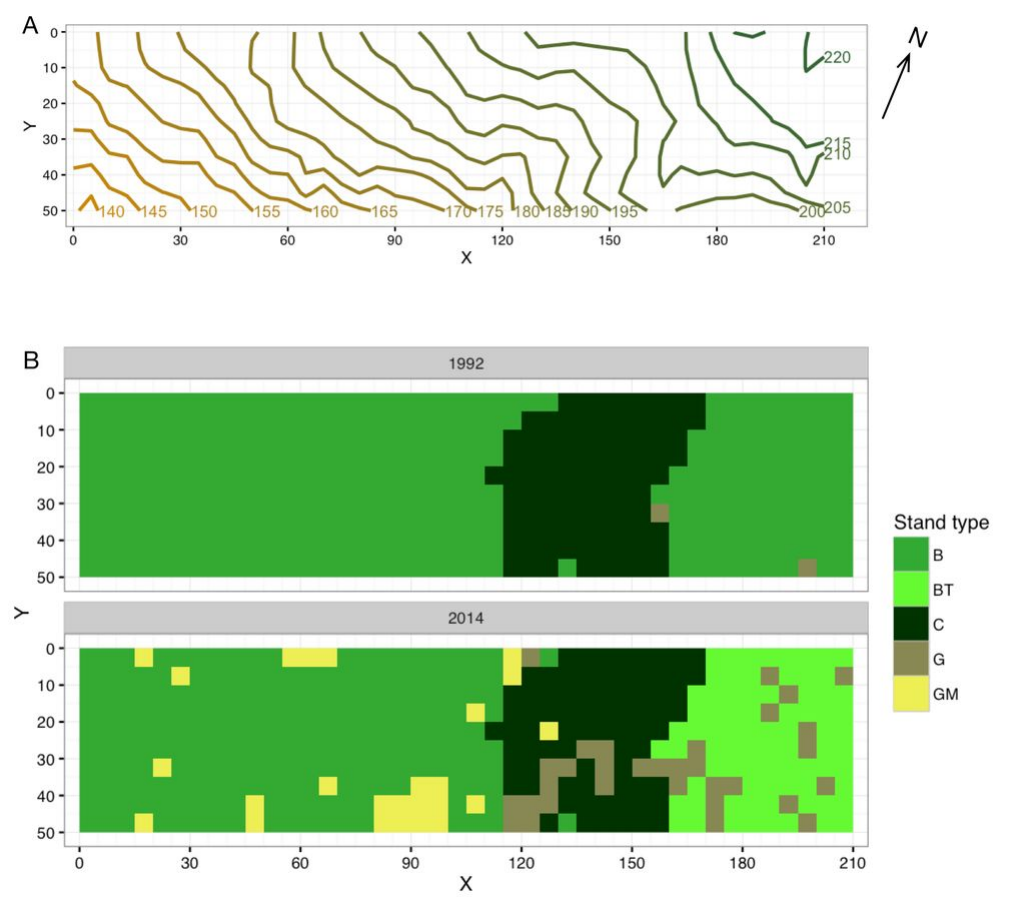
Outline of the study site. A) Contour map. B) Stand type. B: broadleaved, BT: broadleaved (thinned), C: conifer plantation, G: gap (not related to mass mortality of oak trees), GM: gap created or affected by mass mortality of oak trees. A GM in the conifer plantation was generated by a dead oak stem that remained in the plantation.

A 1.05 ha (210 m × 50 m) study site was established in 1992 (Fig. 1). The site was divided into 420 quadrats of 5 × 5 m. The study site mainly consisted of broadleaved secondary forest stands, and the rest was a conifer plantation. The plantation was thinned in 1996, and some of the evergreen broadleaved trees were thinned in the eastern part of the site in 2005. After mass mortality, dead stems of *Q. serrata* were felled, and some stems surrounding the dead stems were also felled. The felled trunks were cut into about 1 m lengths and piled on the floor. The infected wood was covered with plastic sheets and disinfected. The cut stems are denoted in the measurement data file.

Two classes of forest layers were defined: the canopy and sub-canopy layer and the understory layer. Stems in the canopy and sub-canopy layer were defined as having a diameter at breast height (dbh) at least 3.0 cm, and stems in the understory layer were defined as those with a dbh smaller than 3.0 cm or a height shorter than 1.3 m (not including those seedlings and shoots sprouted in the current year). During the period from December 1993 to February 1994, all stems in the canopy and sub-canopy layer were marked, identified by species name, and their dbh measured using a measuring tape with 1 mm precision. After that, dbh was measured in 1996, 1999, 2002, 2005 and 2014 during the non-growing season (October to January of the following year) in the same way. Measurement values in 1999 are missing in the most eastern half of the study site because slope collapse prevention works were conducted near the area.

In 1992, the species of woody plants found in the understory layer were recorded for each quadrat to obtain the understory species composition of the forest in autumn (September to November). The same survey was conducted again in 2014 (July to November). The dynamics of the half of the study site that solely consisted of broadleaved forest was reported in Itô (2015) and Itô (2016), but this is the first report of the whole study site.

## Geographic coverage

### Description

Ginkakuzi-san National Forest, Kyôto City, Japan

### Coordinates

35.028 and 35.029 Latitude; 135.802 and 135.800 Longitude.

## Taxonomic coverage

### Description

The six surveys from 1993 to 2014 found 61 species in the canopy and sub-canopy layer. Table 1 shows their stem density (stems/ha) and basal area (m^2^/ha) in 1993 and 2014. *Clerodendrum trichotomum* Thunb. was not included in Table 1, because it had only one stem, which was first found in 2002 but had disappeared in 2005.

**Figure 2.**
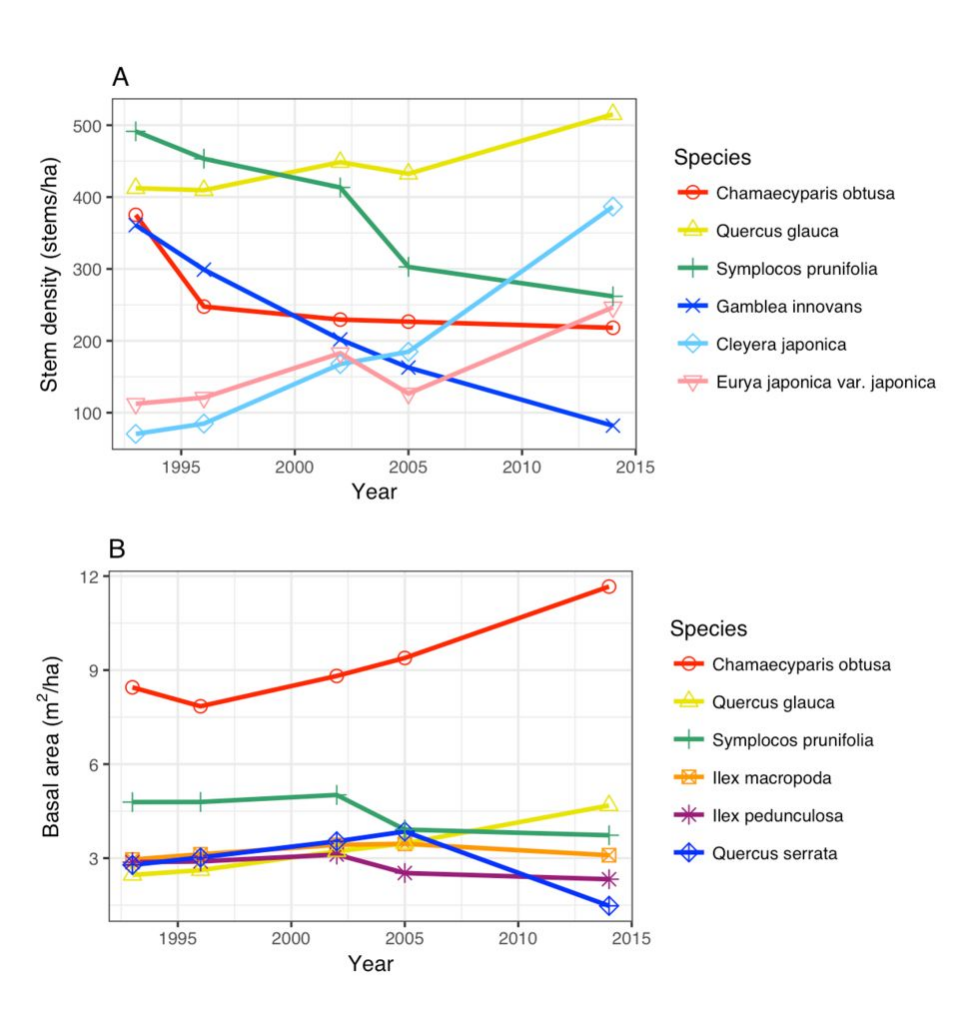
Changes in dominance of major species. A: stem density, and B: basal area.

The most abundant species was Symplocos prunifolia Siebold & Zucc. in 1993, but *Quercus glauca* Thunb. surpassed it by 2014. The dominant species in the basal area was consistently Chamaecyparis obtusa, which occupied most of the plantation area of the study site. *Quercus serrata* decreased in density from 37.1 to 14.3 stems/ha and in basal area from 2.8 to 1.5 m^2^/ha (Table 1, Fig. 2).

In the understory layer, 88 woody species were found over the two surveys in 1992 and 2014 excluding unidentified species. Table 2 shows the species and the number of quadrats where they were found. *Quercus glauca* was the most frequent species in both 1992 and 2014. *Symplocos prunifolia* increased in the number of quadrats from 75 to 274. This may related to gap formation by oak tree deaths and thinning of upper trees as well as the deer-unpalatable trait of the species.

## Temporal coverage

**Data range:** 1992-9-18 – 2014-12-19.

## Usage rights

### Use license

Open Data Commons Attribution License

### IP rights notes

Forestry and Forest Products Research Institute (Matsunosato 1, Tsukuba 305-8687, Japan) has ownership of this data set.

## Data resources

### Data package title

Forest dynamics data in the Ginkakuzi-san National Forest, Kyôto, Japan

### Number of data sets

6

### Data set 1.

#### Data set name

Site data

#### Data format

CSV

#### Number of columns

4

#### Download URL

http://dx.doi.org/10.5061/dryad.7f399

#### Description

Location and stand type of each quadrat.

**Data set 1.** 

Column label	Column description
X	Position of the northwest corner of the quadrat along the X axis (m).
Y	Position of the northwest corner of the quadrat along the Y axis (m).
Type1992	Stand type of the quadrat (B: broadleaved forest, C: conifer plantation, G: gap).
Type2014	Stand type of the quadrat (B: broadleaved forest, C: conifer plantation, G: gap (not related to mass mortality of oak trees), GM: gap created or affected by the mass mortality of oak trees).

### Data set 2.

#### Data set name

Elevation data

#### Data format

CSV

#### Number of columns

3

#### Download URL

http://dx.doi.org/10.5061/dryad.7f399

#### Description

Elevation of grid points (5 m × 5 m) of the study site.

**Data set 2.** 

Column label	Column description
X	Location along the X axis (m).
Y	Location along the Y axis (m).
Elevation	Elevation (m; precision: 0.1 m).

### Data set 3.

#### Data set name

Stem data

#### Data format

CSV

#### Number of columns

9

#### Download URL

http://dx.doi.org/10.5061/dryad.7f399

#### Description

List of all stems found from 1993 to 2014.

**Data set 3.** 

Column label	Column description
Indv	Individual ID
Stem	Stem ID
X	Position of the northwest corner of the quadrat where the stem was located along the X axis (m).
Y	Position of the northwest corner of the quadrat where the stem was located along the Y axis (m).
X1	Position of the stem along the X axis (m; precision: 0.1 m).
Y1	Position of the stem along the X axis (m; precision: 0.1 m).
Species	Species of the stem.
Start	Year when the stem was first marked.
End	Year when the stem was last found alive (NA denotes that the stem was still alive in 2014).

### Data set 4.

#### Data set name

Stem measurement data

#### Number of columns

4

#### Download URL

http://dx.doi.org/10.5061/dryad.7f399

#### Description

Measurements of dbh for each stem from 1993 to 2014. Measurement values in 1999 are missing in the most eastern half of the study site because slope collapse prevention works were conducted near the area.

**Data set 4.** 

Column label	Column description
Stem	Stem ID
Year	Year of the measurement.
DBH	Diameter at breast height (cm; precision: 0.1 cm). NA denotes missing data.
Comment	Comment on the measurement.

### Data set 5.

#### Data set name

Understory data

#### Data format

CSV

#### Number of columns

4

#### Download URL

http://dx.doi.org/10.5061/dryad.7f399

#### Description

Occurrence of woody species in the understory layer for each quadrat.

**Data set 5.** 

Column label	Column description
Year	Survey year.
X	Position of the northwest corner of the quadrat along the X axis (m).
Y	Position of the northwest corner of the quadrat along the Y axis (m).
Species	Species found in the quadrat.

### Data set 6.

#### Data set name

Occurrence data of woody species

#### Data format

Darwin Core Archive

#### Number of columns

23

#### Download URL

http://www.gbif.org/dataset/d5d92045-cbd8-453a-9b4e-25a7b74c51c5

#### Description

Occurrence data of woody species in the Ginkakuzi-san National Forest.

**Data set 6.** 

Column label	Column description
occurrenceID	An identifier for the Occurrence.
modified	The most recent date-time on which the resource was changed.
rights	Information about who can access the resource or an indication of its security status.
rightsHolder	A person or organization owning or managing rights over the resource.
institutionCode	The name (or acronym) in use by the institution having custody of the object(s) or information referred to in the record.
collectionCode	The name, acronym, coden, or initialism identifying the collection or data set from which the record was derived.
datasetName	The name identifying the data set from which the record was derived.
basisOfRecord	The specific nature of the data record.
catalogNumber	An identifier (preferably unique) for the record within the data set or collection.
year	The four-digit year in which the Event occurred, according to the Common Era Calendar.
country	The name of the country or major administrative unit in which the Location occurs.
countryCode	The standard code for the country in which the Location occurs.
verbatimLocality	The original textual description of the place.
decimalLatitude	The geographic latitude (in decimal degrees, using the spatial reference system given in geodeticDatum) of the geographic center of a Location.
decimalLongitude	The geographic longitude (in decimal degrees, using the spatial reference system given in geodeticDatum) of the geographic center of a Location.
coordinateUncertaintyInMeters	The horizontal distance (in meters) from the given decimalLatitude and decimalLongitude describing the smallest circle containing the whole of the Location.
scientificName	The full scientific name, with authorship and date information if known.
kingdom	The full scientific name of the kingdom in which the taxon is classified.
phylum	The full scientific name of the phylum or division in which the taxon is classified.
family	The full scientific name of the family in which the taxon is classified.
genus	The full scientific name of the genus in which the taxon is classified.
specificEpithe	The name of the first or species epithet of the scientificName.
infraspecificEpithet	The name of the lowest or terminal infraspecific epithet of the scientificName, excluding any rank designation.

## Figures and Tables

**Table 1.** Changes in number of stems and basal area for each species in the canopy and sub-canopy layer.

Species	D1993	D2014	B1993	B2014
*Chamaecyparis obtusa* (Siebold & Zucc.) Endl.	375.2	218.1	8.5E+00	1.2E+01
*Quercus glauca* Thunb.	412.4	515.2	2.5E+00	4.7E+00
*Symplocos prunifolia* Siebold & Zucc.	491.4	261.9	4.8E+00	3.7E+00
*Cryptomeria japonica* (L.f.) D.Don	81.9	61.9	2.3E+00	3.2E+00
*Ilex macropoda* Miq.	138.1	103.8	3.0E+00	3.1E+00
*Ilex pedunculosa* Miq.	193.3	141.0	2.9E+00	2.3E+00
*Gamblea innovans* (Siebold & Zucc.) C.B.Shang, Lowry & Frodin	361.0	81.9	3.0E+00	1.5E+00
*Quercus serrata* Murray	37.1	14.3	2.8E+00	1.5E+00
*Chengiopanax sciadophylloides* (Franch. & Sav.) C.B.Shang et J.Y.Huang	78.1	20.0	1.5E+00	8.1E-01
*Cleyera japonica* Thunb.	70.5	386.7	8.7E-02	8.1E-01
*Carpinus laxiflora* (Siebold & Zucc.) Blume	29.5	18.1	7.6E-01	7.8E-01
*Cerasus jamasakura* (Siebold ex Koidz.) H.Ohba	12.4	6.7	3.4E-01	6.3E-01
*Photinia glabra* (Thunb.) Maxim.	79.0	85.7	4.1E-01	5.3E-01
*Carpinus tschonoskii* Maxim.	8.6	5.7	3.7E-01	5.1E-01
*Wisteria floribunda* (Willd.) DC.	119.0	90.5	3.3E-01	4.9E-01
*Padus grayana* (Maxim.) C.K.Schneid.	17.1	10.5	2.6E-01	3.9E-01
*Abies firma* Siebold & Zucc.	3.8	2.9	1.8E-01	3.4E-01
Eurya japonica Thunb. var. japonica	112.4	246.7	1.2E-01	3.3E-01
Lyonia ovalifolia (Wall.) Drude var. elliptica (Siebold & Zucc.) Hand.-Mazz.	61.9	19.0	3.9E-01	2.3E-01
*Albizia julibrissin* Durazz.	13.3	1.9	4.1E-01	2.2E-01
*Aria japonica* Decne.	1.0	1.0	1.2E-01	1.6E-01
*Acer palmatum* Thunb.	2.9	6.7	1.2E-01	1.5E-01
*Ilex chinensis* Sims	1.9	1.9	2.2E-01	1.4E-01
*Fraxinus sieboldiana* Blume	22.9	10.5	2.0E-01	1.3E-01
*Mallotus japonicus* (L.f.) Müll.Arg.	4.8	1.9	1.5E-01	1.3E-01
*Camellia japonica* L.	25.7	27.6	5.6E-02	1.2E-01
*Styrax japonica* Siebold & Zucc.	35.2	9.5	3.0E-01	1.2E-01
*Clethra barbinervis* Siebold & Zucc.	22.9	10.5	2.5E-01	1.0E-01
*Dendropanax trifidus* (Thunb.) Makino ex H.Hara	4.8	4.8	8.8E-02	9.9E-02
*Cinnamomum camphora* (L.) J.Presl	1.0	3.8	7.8E-03	9.6E-02
*Ilex rotunda* Thunb.	1.9	1.9	5.2E-02	8.8E-02
*Diospyros kaki* Thunb.	5.7	1.9	8.3E-02	8.2E-02
*Laurocerasus spinulosa* (Siebold & Zucc.) C.K.Schneid.	3.8	1.9	2.0E-01	8.0E-02
*Ligustrum japonicum* Thunb.	15.2	17.1	3.3E-02	6.4E-02
*Idesia polycarpa* Maxim.	1.9	1.0	2.2E-02	6.3E-02
*Alnus sieboldiana* Matsum.	2.9	1.0	1.9E-01	6.2E-02
*Zanthoxylum ailanthoides* Siebold & Zucc.	3.8	1.9	1.8E-01	4.8E-02
*Castanopsis cuspidata* (Thunb.) Schottky	1.0	10.5	1.6E-02	4.7E-02
*Vaccinium bracteatum* Thunb.	17.1	4.8	3.6E-02	3.9E-02
*Euscaphis japonica* (Thunb.) Kanitz	6.7	1.0	8.0E-03	2.5E-02
*Toxicodendron sylvestre* (Siebold & Zucc.) Kuntze	2.9	1.0	3.4E-02	1.1E-02
*Magnolia compressa* Maxim.	0.0	1.0	0.0E+00	3.7E-03
*Rhododendron reticulatum* D.Don ex G.Don	9.5	3.8	9.2E-03	3.6E-03
*Osmanthus heterophyllus* (G.Don) P.S.Green	3.8	2.9	4.9E-03	3.5E-03
*Pieris japonica* (Thunb.) D.Don ex G.Don	5.7	1.0	1.1E-02	3.2E-03
*Symplocos sawafutagi* Nagam.	1.9	2.9	1.6E-03	3.2E-03
Pourthiaea villosa (Thunb.) Decne. var. villosa	1.0	1.0	8.6E-04	2.5E-03
*Triadica sebifera* (L.) Small	0	1.0	0.0E+00	2.2E-03
*Acer crataegifolium* Siebold & Zucc.	1.9	0	3.9E-03	0.0E+00
*Alnus firma* Siebold & Zucc.	1.0	0	2.3E-03	0.0E+00
*Amelanchier asiatica* (Siebold & Zucc.) Endl. ex Walp.	2.9	0	1.1E-02	0.0E+00
Aucuba japonica Thunb. var. japonica	1.0	0	9.7E-04	0.0E+00
*Castanea crenata* Siebold & Zucc.	1.9	0	3.0E-02	0.0E+00
*Cinnamomum yabunikkei* H.Ohba	1.0	0	1.7E-03	0.0E+00
*Elaeagnus glabra* Thunb.	1.0	0	2.3E-03	0.0E+00
*Ilex crenata* Thunb.	1.0	0	8.6E-04	0.0E+00
*Ilex micrococca* Maxim.	1.0	0	1.3E-01	0.0E+00
*Pinus densiflora* Siebold & Zucc.	20.0	0	1.5E+00	0.0E+00
*Quercus acutissima* Carruth.	1.0	0	1.7E-03	0.0E+00
*Toxicodendron trichocarpum* (Miq.) Kuntze	23.8	0	5.8E-02	0.0E+00
Total	2961.4	2428.3	3.91E+01	3.99E+01

**Table 2.** Changes in number of quadrats where each species was found (out of 420 quadrats) in the understory layer.

Species	1992	2014
*Quercus glauca* Thunb.	361	392
Eurya japonica Thunb. var. japonica	362	304
*Symplocos prunifolia* Siebold & Zucc.	75	274
*Cryptomeria japonica* (L.f.) D.Don	190	163
*Photinia glabra* (Thunb.) Maxim.	168	114
*Cryptomeria japonica* (L.f.) D.Don	26	79
*Ilex pedunculosa* Miq.	36	72
*Carpinus laxiflora* (Siebold & Zucc.) Blume	15	58
*Chamaecyparis obtusa* (Siebold & Zucc.) Endl.	13	58
*Styrax japonica* Siebold et Zucc.	4	53
*Camellia japonica* L.	64	51
*Mallotus japonicus* (L.f.) Müll.Arg.	2	46
*Ilex macropoda* Miq.	6	45
*Cleyera japonica* Thunb.	7	44
*Celtis sinensis* Pers.	0	40
*Zanthoxylum ailanthoides* Siebold & Zucc.	0	37
*Osmanthus heterophyllus* (G.Don) P.S.Green	26	35
*Carpinus tschonoskii* Maxim.	1	29
*Fraxinus sieboldiana* Blume	0	28
*Callicarpa mollis* Siebold & Zucc.	13	25
*Castanopsis cuspidata* (Thunb.) Schottky	19	22
*Ilex micrococca* Maxim.	0	22
*Zelkova serrata* (Thunb.) Makino	2	20
*Gamblea innovans* (Siebold & Zucc.) C.B.Shang, Lowry & Frodin	24	18
*Aphananthe aspera* (Thunb.) Planch.	1	18
*Pinus densiflora* Siebold & Zucc.	2	17
*Cinnamomum yabunikkei* H.Ohba	26	16
*Quercus serrata* Murray	14	16
*Ilex crenata* Thunb.	143	15
*Abelia serrata* Siebold & Zucc.	17	15
*Acer palmatum* Thunb.	9	15
*Chengiopanax sciadophylloides* (Franch. & Sav.) C.B.Shang & J.Y.Huang	13	14
*Triadica sebifera* (L.) Small	0	14
*Ligustrum japonicum* Thunb.	68	13
*Aria japonica* Decne.	2	12
*Idesia polycarpa* Maxim.	0	12
*Rubus microphyllus* L.f.	0	11
*Laurocerasus spinulosa* (Siebold & Zucc.) C.K.Schneid.	20	10
*Pieris japonica* (Thunb.) D.Don ex G.Don	12	10
*Rhododendron reticulatum* D.Don ex G.Don	4	7
*Albizia julibrissin* Durazz.	0	7
*Cinnamomum camphora* (L.) J.Presl	0	7
*Rhododendron macrosepalum* Maxim.	26	6
*Cornus macrophylla* Wall.	0	6
Aucuba japonica Thunb. var. japonica	211	5
*Vaccinium bracteatum* Thunb.	61	5
*Toxicodendron trichocarpum* (Miq.) Kuntze	42	5
Lyonia ovalifolia (Wall.) Drude var. elliptica (Siebold & Zucc.) Hand.-Mazz.	20	5
*Abies firma* Siebold & Zucc.	3	5
*Symplocos sawafutagi* Nagam.	2	5
*Ardisia crenata* Sims	3	4
*Rubus buergeri* Miq.	64	3
*Lindera umbellata* Thunb.	39	3
*Elaeagnus pungens* Thunb.	4	3
Rubus palmatus Thunb. var. palmatus	1	3
*Rubus phoenicolasius* Maxim.	0	3
*Acer crataegifolium* Siebold & Zucc.	4	2
*Padus grayana* (Maxim.) C.K.Schneid.	3	2
Damnacanthus indicus C.F.Gaertn. var. indicus	2	2
*Aralia elata* (Miq.) Seem.	0	2
*Cerasus jamasakura* (Siebold ex Koidz.) H.Ohba	0	2
*Ilex chinensis* Sims	0	2
*Rosa multiflora* Thunb.	0	2
*Rubus hirsutus* Thunb.	0	2
Unidentified	0	2
*Vaccinium hirtum* Thunb.	6	1
*Vaccinium smallii* A.Gray	6	1
*Castanea crenata* Siebold & Zucc.	2	1
*Clerodendrum trichotomum* Thunb.	2	1
*Illicium anisatum* L.	1	1
*Broussonetia monoica* Hance	0	1
*Epigaea asiatica* Maxim.	0	1
*Ilex integra* Thunb.	0	1
*Zanthoxylum piperitum* (L.) DC.	0	1
*Viburnum erosum* Thunb.	11	0
*Ardisia japonica* (Thunb.) Blume	10	0
*Trachycarpus fortunei* (Hook.) H.Wendl.	7	0
*Euscaphis japonica* (Thunb.) Kanitz	6	0
Rhododendron kaempferi Planch. var. kaempferi	6	0
*Dendropanax trifidus* (Thunb.) Makino ex H.Hara	4	0
*Neolitsea sericea* (Blume) Koidz.	4	0
*Camellia sinensis* (L.) Kuntze	3	0
*Carpinus* sp.	2	0
*Diospyros kaki* Thunb.	2	0
*Neolitsea* sp.	2	0
Pourthiaea villosa (Thunb.) Decne. var. villosa	2	0
Cephalotaxus harringtonia (Knight ex Forbes) K.Koch var. harringtonia	1	0
*Elaeagnus* sp.	1	0
*Fatsia japonica* (Thunb.) Decne. & Planch.	1	0
*Quercus acutissima* Carruth.	1	0
*Symplocos lancifolia* Siebold & Zucc.	1	0
*Vaccinium japonicum* Miq.	1	0
Vaccinium sp.	1	0

## References

[B3527908] Côté Steeve D., Rooney Thomas P., Tremblay Jean-Pierre, Dussault Christian, Waller Donald M. (2004). Ecological Impacts of Deer Overabundance. Annual Review of Ecology, Evolution, and Systematics.

[B3527920] Gerhardt Philipp, Arnold Johanna Maria, Hackländer Klaus, Hochbichler Eduard (2013). Determinants of deer impact in European forests – A systematic literature analysis. Forest Ecology and Management.

[B3527930] Itô Hiroki (2007). Twelve years change of a broad leaved secondary forest in Ginkakuji-san National Forest. Bulletin of FFPRI.

[B3527940] Itô Hiroki (2015). Effects of Sika Deer （*Cervus
nippon*） on the Dynamics of a Broadleaved Secondary Forest after Mass Mortality of Oak Trees. Journal of the Japanese Forest Society.

[B3527951] Itô Hiroki (2016). Changes in understory species occurrence of a secondary broadleaved forest after mass mortality of oak trees under deer foraging pressure. PeerJ.

[B3577905] Kinuura Haruo, Kobayashi Masahide (2006). Death of Quercus
crispula by inoculation with adult Platypus
quercivorus (Coleoptera: Platypodidae). Applied Entomology and Zoology.

[B3577895] Kubono Takanori, Ito Shin-ichiro (2002). Raffaelea
quercivora sp. nov. associated with mass mortality of Japanese oak, and the ambrosia beetle (Platypus
quercivorus). Mycoscience.

[B3527961] Kuroda K., Osumi K., Oku H. (2012). Reestablishing the health of secondary forests “Satoyama” endangered by Japanese oak wilt: A preliminary report. Journal of Agricultural Extension and Rural Development.

[B3577915] Mamiya Y (1988). History of pine wilt disease in Japan.. Journal of nematology.

[B3527971] Müllerová Jana, Hédl Radim, Szabó Péter (2015). Coppice abandonment and its implications for species diversity in forest vegetation. Forest Ecology and Management.

[B3527981] Nakajima Haruki, Ishida Megumi (2014). Decline of *Quercus
crispula* in abandoned coppice forests caused by secondary succession and Japanese oak wilt disease: Stand dynamics over twenty years. Forest Ecology and Management.

[B3528047] Ogura Jun'ich, Nature and Environmental Conservation Division Department of the Environment of the Kyoto Prefecture (2015). *Satotyama* vegetation landscape in the southern part of the Kyoto Prefecture in the Meiji Era. Red Data Book of the Kyoto Prefecture.

[B3528083] Office Osaka Regional Forest (1936). Landscape planning of the Higashiayama National Forest.

[B3528003] Rackham Oliver (2008). Ancient woodlands: modern threats. New Phytologist.

[B3528068] Takahara Hikaru, Nature and Environmental Conservation Division Department of the Environment of the Kyoto Prefecture (2015). Vegetation history of Kyoto Prefecture after the last interglacial period. Red Data Book of the Kyoto Prefecture.

[B3528013] Takatsuki Seiki (2009). Effects of sika deer on vegetation in Japan: A review. Biological Conservation.

[B3528023] Takeuchi Kazuhiko (2010). Rebuilding the relationship between people and nature: the Satoyama Initiative. Ecological Research.

[B3528033] Washitani Izumi (2001). Traditional sustainable ecosystem ‘SATOYAMA’ and biodiversity crisis in Japan: Conservation ecological perspective. Global Environment Research.

